# Development and characterization of microsatellite loci in *Grevillea robusta*

**DOI:** 10.1186/1753-6561-5-S7-P16

**Published:** 2011-09-13

**Authors:** Camila Mantello, Daiane Rigoni Kestring, Valderês Aparecida Sousa, Ananda Virginia Aguiar, Anete Pereira Souza

**Affiliations:** 1 UNICAMP, Brazil; 2 Embrapa Florestas, Brazil

## Background

*Grevillea robusta* is a native tree to the subtropical coastal regions of northern New South Wales and southern Queensland in Australia. In Brazil, Embrapa Forestry and its partners have established many provenance/progeny tests to increase the species genetic basis and aim to implement a breeding program to improve timber production. Genetic variability of these tests has been monitored through the assessment of quantitative traits. However, the genetic evaluation of materials based on phenotypic traits is influenced by many environmental factors. Estimates of some genetic parameters such as gene flow and parentage are possible using molecular tools as most common molecular marker, microsatellite (Simple Sequence Repeats, SSRs) which are codominant and highly polymorphic. Genetic markers have intensively applied for the main strategies in breeding programs, especially when economically important traits are difficult measure because of low heritability. The aim of this study was develop microsatellite markers for *Grevillea robusta* through enriched library in order to estimate the genetic diversity and structure of the species, and direct efforts for the conservation and management of its active germplasm banks.

## Methods

The genomic-enriched library was constructed following the protocol described by [1]. The genomic DNA was digested with AFA*I* and enriched in (CT)_8_ and (GT)_8_ repeats. Enriched fragments were amplified by polymerase chain reaction (PCR), connected to a pGEM T-easy vector and transformed into competent XL1- blue *Escherichia coli* cells. The positive clones were selected using the B-galactosidase gene and then grown overnight in an HM/F medium with ampicillin. After PCR, 95 positive clones were sequenced in both directions using the T7 and SP6 primers as well as the Big Dye terminator Kit. The sequences were assembled and edited in Seqman (DNAStar) and the repetitive regions were found using the Simple Sequence Repeat Identification Tool [2]. Primer Select (DNAStar) and Primer 3 plus were softwares used to design primer pairs flanking the microsatellite regions.

## Results and conclusions

Of the ninety five sequences cloned, seven contained microsatellite sequences. Only five of them showed repeats and adequate flanking regions for primer design. The observed proportion of trinucleotide was 4.21% (4), while tetranucleotide and pentanucleotide were 2.11% (2) and 1.05% (1), respectively. Of the total of nucleotides found 100% are simply perfect. The explanation for this low yield sequences (7,4%) can be attributed to the genomic-enriched procedure. These sequences will be validated and promptly used to estimate the genetic diversity, gene flow and parentage of the Brazilian germoplasm collections.
